# Topical Administration of a Soluble TNF Inhibitor Reduces Infarct Volume After Focal Cerebral Ischemia in Mice

**DOI:** 10.3389/fnins.2019.00781

**Published:** 2019-08-07

**Authors:** Minna Yli-Karjanmaa, Bettina Hjelm Clausen, Matilda Degn, Hans Gram Novrup, Ditte Gry Ellman, Peter Toft-Jensen, David E. Szymkowski, Allan Stensballe, Morten Meyer, Roberta Brambilla, Kate Lykke Lambertsen

**Affiliations:** ^1^Department of Neurobiology Research, Institute of Molecular Medicine, University of Southern Denmark, Odense, Denmark; ^2^BRIDGE – Brain Research Inter-Disciplinary Guided Excellence, Department of Clinical Research, University of Southern Denmark, Odense, Denmark; ^3^Pediatric Oncology Laboratory, Department of Pediatrics and Adolescent Medicine, University Hospital Rigshospitalet, Copenhagen, Denmark; ^4^Xencor Inc., Monrovia, CA, United States; ^5^Department of Health Science and Technology, University of Aalborg, Aalborg, Denmark; ^6^The Miami Project to Cure Paralysis, University of Miami Miller School of Medicine, Miami, FL, United States; ^7^Department of Neurology, Odense University Hospital, Odense, Denmark

**Keywords:** ischemic stroke, behavior, cytokines, microglial activation, neuroprotection

## Abstract

**Background:**

Tumor necrosis factor, which exists both as a soluble (solTNF) and a transmembrane (tmTNF) protein, plays an important role in post-stroke inflammation. The objective of the present study was to test the effect of topical versus intracerebroventricular administration of XPro1595 (a solTNF inhibitor) and etanercept (a solTNF and tmTNF inhibitor) compared to saline on output measures such as infarct volume and post-stroke inflammation in mice.

**Methods:**

Adult male C57BL/6 mice were treated topically (2.5 mg/ml/1μl/h for 3 consecutive days) or intracerebroventricularly (1.25 mg/kg/0.5 ml, once) with saline, XPro1595, or etanercept immediately after permanent middle cerebral artery occlusion (pMCAO). Mice were allowed to survive 1 or 3 days. Infarct volume, microglial and leukocyte profiles, and inflammatory markers were evaluated.

**Results:**

We found that topical, and not intracerebroventricular, administration of XPro1595 reduced infarct volume at both 1 and 3 days after pMCAO. Etanercept showed no effect. We observed no changes in microglial or leukocyte populations. XPro1595 increased gene expression of *P2ry12* at 1 day and *Trem2* at 1 and 3 days, while decreasing *Cx3cr1* expression at 1 and 3 days after pMCAO, suggesting a change in microglial activation toward a phagocytic phenotype.

**Conclusion:**

Our data demonstrate that topical administration of XPro1595 for 3 consecutive days decreases infarct volumes after ischemic stroke, while modifying microglial activation and the inflammatory response post-stroke. This suggests that inhibitors of solTNF hold great promise for future neuroprotective treatment in ischemic stroke.

## Introduction

Inflammatory events after ischemic stroke contribute to secondary injury mechanisms that can expand tissue injury and are thus targets of new treatment options (Lambertsen et al., 2018). Tumor necrosis factor (TNF) is a multifunctional, proinflammatory cytokine that participates in all phases of ischemic stroke, from development to repair and long-term inflammatory effects (reviewed in Hallenbeck, 2002). It exists both as a transmembrane (tmTNF) and a soluble (solTNF) protein, the latter released via proteolytic cleavage by TNFα converting enzyme (TACE, also called ADAM17) (Black et al., 1997). TNF mediates its effects via TNF receptor 1 (TNFR1, p55TNFR) and 2 (TNFR2, p75TNFR); solTNF primarily mediates acute and chronic inflammation, while tmTNF has been shown to support innate immune function and promote neuronal survival and axonal remyelination, synaptic function, and neurotransmission (Ruuls et al., 2001; Alexopoulou et al., 2006; Brambilla et al., 2011; Barnum et al., 2014; Cavanagh et al., 2016). In ischemic stroke, TNF plays an important role in infarct development (Lambertsen et al., 2018). In support of this, conventional (Lambertsen et al., 2009) and conditional myeloid (Clausen et al., 2016a) deletion of TNF resulted in increased infarct volumes in mice subjected to permanent middle cerebral artery occlusion (pMCAO). Interestingly, genetic ablation of solTNF using tmTNF^Δ/Δ^ mice resulted in decreased infarct volumes after pMCAO (Madsen et al., 2016), suggesting that inhibition of solTNF, but preservation of tmTNF, is required for neuroprotection in ischemic stroke.

Current FDA- and EMA-approved TNF inhibitors are used for treating chronic autoimmune inflammatory diseases such as rheumatoid arthritis, inflammatory bowel disease, and psoriasis (Lis et al., 2014), where they also relieve potential symptoms of depression (Tyring et al., 2006). The dimeric Fc-fusion protein etanercept targets both tmTNF and solTNF. Being a large (150 kDa) protein, it is not able to cross the blood-brain barrier. When administered perispinally, however, etanercept improved neurological outcome in patients with chronic stroke, traumatic brain injury and Alzheimer’s disease (Griffin, 2008; Tobinick, 2011; Tobinick et al., 2012; and reviewed in Lambertsen et al., 2018). The downside of drugs like etanercept is that inhibition of both solTNF and tmTNF can provoke demyelination and induce neuropathies, infections, sepsis, congestive heart failure and lupus, emphasizing the importance of leaving tmTNF available to preserve adequate function of the innate immune system (Scheinfeld, 2004). XPro1595 is a dominant-negative inhibitor of solTNF (Steed et al., 2003). In animal models of multiple sclerosis, XPro1595 promotes axonal preservation and remyelination (Brambilla et al., 2011); in Parkinson’s disease XPro1595 reduces neuronal loss (McCoy et al., 2006; Barnum et al., 2014); in Alzheimer’s disease it decreases beta-amyloid plaques and promotes synaptic function (Cavanagh et al., 2016; MacPherson et al., 2017); and in spinal cord injury it decreases lesion volume (Novrup et al., 2014). We have previously shown that using etanercept and XPro1595 for systemic inhibition of TNF improved functional outcome and affected the inflammatory response after pMCAO in mice, but with no effect on infarct volume (Clausen et al., 2014). The altered inflammatory response after systemic administration of TNF inhibitors was likely a result of peripheral immunomodulation that decreased the granulocyte infiltration to the ischemic lesion, which was earlier shown to be neuroprotective (Soriano et al., 1999; Clausen et al., 2014). Peripheral treatment likely also changed microglial activation (Clausen et al., 2014). In light of these studies and our earlier studies demonstrating that topical (and not systemic) administration of XPro1595 decreased lesion size following moderate spinal cord injury (Novrup et al., 2014), we hypothesized that topical and/or intracerebroventricular (i.c.v.) delivery of XPro1595 would reduce lesion size, ameliorate brain inflammation, and improve functional outcome after focal cerebral ischemia in mice.

## Materials and Methods

### Animals

Adult male C57BL/6 mice (aged 7–8 weeks) were purchased from Taconic Ltd. (Ry, Denmark) and transferred to the Biomedical Laboratory at the University of Southern Denmark, Odense, Denmark. tmTNF^Δ/Δ^ (Ruuls et al., 2001) breeding couples were kindly donated by Dr. Tacchini-Cottier, University of Lausanne, Switzerland and a breeding colony was established at the Biomedical Laboratory at the University of Southern Denmark. Animals were housed under diurnal lighting conditions, and food and water were available *ad libitum.* Mice were allowed to acclimatize for at least 1 week before behavioral testing. All animal experiments were approved by the Danish Animal Inspectorate under the Ministry of Food and Agriculture (J. No. 2013-15-2934-00924).

### Permanent Middle Cerebral Artery Occlusion

Permanent occlusion of the distal part of the middle cerebral artery was performed by electrocoagulation under Hypnorm-Dormicum anesthesia (fentanyl citrate (0.315 mg/ml, Jansen-Cilag), fluanisone (10 mg/ml, Jansen-Cilag, Birkerød, Denmark), and midazolam (5 mg/ml, Hoffmann-La Roche, Hvidovre, Denmark) as routinely done in our laboratory (Lambertsen et al., 2001, 2009; Clausen et al., 2014, 2016b). Post-operatively, mice were injected with 0.9% physiological saline subcutaneously (s.c.) and placed in a 28°C heating cabinet for 24 h. Mice with 3 days survival were returned to the conventional animal facility after 24 h. Buprenorphine hydrochloride (0.001 mg/20 g Temgesic, Schering-Plough, Ballerup, Denmark) was administered three times at 8 h intervals starting immediately prior to surgery. Sham mice were subjected to similar surgery but without electrocoagulation of the middle cerebral artery (MCA).

### Pharmacological Treatment

Saline, XPro1595 (Xencor Inc., Monrovia, CA, United States), or etanercept (Enbrel, Amgen-Wyeth, Thousand Oak, CA, United States) was administered topically using mini-osmotic pumps (Alzet, 1003D, Durect Corporation, Cupertino, CA, United States) implanted 30 min after pMCAO. XPro1595 and etanercept were diluted in saline for the final concentration. The pumps were placed in such a way that the delivering end of the catheter was on top of the infarct core. The catheter was sutured to the musculature, and the suture and placement of it were secured using Vetbond (3M Animal Care Products, St. Paul, MN, United States). The pumps were set to deliver either saline (0.9% physiological NaCl) with a flow of 1 μl/h, XPro1595 or etanercept with a flow of 2.5 mg/ml/1μl/h for 3 consecutive days, as previously described (Novrup et al., 2014).

In addition, intracerebroventricular (i.c.v.) injection of saline, XPro1595, or etanercept was performed 30 min after pMCAO. For i.c.v. delivery, animals were fixed in a stereotactic frame (David Kopf Instruments, United States) immediately after pMCAO, and anesthesia was maintained with isoflurane (2% isoflurane in O_2_). One single i.c.v. injection (0.5 μl) of either saline, XPro1595 (1.25 mg/kg), or etanercept (1.25 mg/kg) was administered using a 2 μl Hamilton micro-syringe. The injection was made in the left lateral ventricle using the following coordinates with reference to bregma: anterior -0.2 mm; lateral 0.9 mm; ventral 2.5 mm; tooth-bar -1.0 mm. After injection, the syringe was left in place for a further 5 min before being slowly retracted.

### Group Size and Study Design

Groups of mice for infarct volumetric, functional, and inflammatory analyses consisted of mice treated topically for 1 day (*n* = 7–11/group) or for 3 days (*n* = 20/group) or mice treated i.c.v. for 1 day (*n* = 6–7/group) or for 3 days (*n* = 20/group) after pMCAO. Animals were excluded from the study if the infarct volume was less than 3 mm^3^ due to lack of successful occlusion of the MCA. Animals with MCA bleedings were excluded due to hematoma formation. A total of 29 mice subjected to pMCAO were excluded due to lack of infarct or bleedings.

To evaluate microglial and leukocyte profiles, a group of sham-treated mice were allowed to survive 3 days (*n* = 5/group). Mice allowed to survive for 1 day (*n* = 5–6/group) or 3 days (*n* = 5/group) after pMCAO were included for flow cytometric studies.

Mortality was 9% and independent of treatment. In total, in the day one groups, 2 mice treated topically with saline, 1 mouse treated topically with XPro1595, 1 mouse treated i.c.v. with XPro1595, and 1 mouse treated i.c.v. with etanercept died. In the day 3 groups, 1 mouse with 3 days survival treated topically with saline, 2 mice treated topically with XPro1595, and 1 mouse treated topically with etanercept, 2 mice treated i.c.v. with saline, 1 mouse treated i.c.v. with XPro1595, and 3 mice treated i.c.v. with etanercept died.

### Behavioral Tests

Functional outcome was evaluated using the Hargreaves test (2 days after surgery) and a grip strength test (3 days after surgery). Prior to behavioral testing, mice were allowed to acclimatize in the behavior room. Neuromuscular testing of mice with mini-osmotic pumps was not possible as the pumps were placed in subcutaneous pockets on the lateral back of the mice and affected the animals’ balance. Behavioral testing was therefore only performed with i.c.v. treated mice.

#### Thermal Nociception Assay

Thermal hyperalgesia was evaluated using the Hargreaves nociception assay (Hargreaves et al., 1988). The plantar test (37370, Ugo Basile, Comerio VA, Italy) was used to measure the withdrawal latencies after predisposing the hind paws to heat (Clausen et al., 2016b). Five measurements were registered for each hind paw prior to and 2 days after pMCAO; the lowest and highest values were excluded, and the mean was calculated. Data are presented as symmetry between contra- and ipsilateral hind paws prior to and after surgery. The mice were allowed to recover for 15 min between trials. Mice were excluded if they did not complete all the trials. In total, four animals were excluded.

#### Grip Strength

Grip strength was measured 1 day prior to and 3 days after surgery using a grip strength meter (BIO-GT-3, BIOSEB, Vitrolles, France). We measured the peak force generated when the mouse loosened its grip when being gently pulled horizontally backwards. We tested both the front right (R) and the front left (L) paw as well as the total grip strength. The peak force (g) of five trials was recorded, and the highest score was registered. Results are presented as delta (Δ) grip strength in grams (g), representing the difference in grip strength prior to and after pMCAO (Lambertsen et al., 2009; Clausen et al., 2014). Mice were excluded if they could not complete the trials with both paws separately and together. A total of four mice were excluded.

### Tissue Processing

#### Fresh Frozen Tissue

Animals were sacrificed by cervical dislocation 1 or 3 days after induction of ischemia. Brains were dissected, frozen in CO_2_ snow, and stored at −80°C until further processing. Brains were cut into six parallel series of 30 μm thick sections as previously described (Clausen et al., 2014).

#### Flow Cytometric Tissue

Mice treated topically with saline, XPro1595, or etanercept were sacrificed 1 or 3 days after pMCAO or 3 days after sham surgery. All mice were given an overdose of pentobarbital (200 mg/ml) containing lidocaine hydrochloride (20 mg/ml) (Glostrup Apotek, Glostrup, Denmark) and perfused through the left ventricle with phosphate buffered saline (PBS, pH 7.4). Ipsilateral and contralateral cortices were dissected and placed in Hanks’ balanced salt solution (HBSS) containing 10% fetal bovine serum (FBS). Lymph nodes were collected for positive detection of T cells. A pool of cells collected from ipsilateral cortices was used for fluorescence minus one (FMO), and isotype controls and a contralateral pool with cells from lymph nodes for compensation. Live/dead cell staining was performed using eBioscience Fixable Viability Dye eFluor 506 (Thermo Fisher Scientific, Roskilde, Denmark).

The tissue was mechanically dissociated using 70 μm cell strainers (BD Biosciences) as previously described (Madsen et al., 2016). Samples were washed with FACS buffer containing HBSS, 2% FBS, and 0.1% sodium azide, fixed with BD Cytofix/Cytoperm (BD Biosciences) and blocked for non-specific binding using 50 μg/ml Syrian Hamster Gamma Globulin (Trichem) and Mouse BD Fc Block (BD Biosciences) diluted in FACS buffer. For detection of microglia and leukocytes, cells were incubated with CD45-PerCP-Cy5.5 (clone 30-F11) and CD11b-PE (clone M7/70).

For separation of macrophages and granulocytes, the following antibodies were used: Ly6C-PE-Cy7 (clone AL21) and Ly6G-BV421 (clone 1A8). T cells were detected with CD3-APC (clone 145-2C11). Respective isotype controls were: IgG2b-PerCP-Cy5.5 (clone A95-1), IgG2b-PE (clone A95-1), IgM-PE-Cy7 (clone R4-22), IgG2a-BV421 (clone R35-95), and IgG1κ-APC (clone A19-3). All antibodies were from BD Biosciences. Flow cytometry was performed as previously described using FACSVerse (BD Biosciences) and analyzed by FACSuite software (Clausen et al., 2014). Positive staining for CD45^dim^CD11b^+^ microglia, CD45^high^CD11b^+^LY6C^+^Ly6G^–^ macrophages, CD45^+^CD3^+^ T cells, and CD45^high^CD11b^+^Ly6C^+^Ly6G^+^ granulocytes was determined based on FMO controls and intensity of the respective isotype control as previously described (Clausen et al., 2016a; Madsen et al., 2016). A total of 1,000,000 events were collected using side scatter (SSC). The mean fluorescence intensity (MFI) was calculated as the geometric mean of each population.

#### Infarct Volumetric and Rostrocaudal Distribution Analysis

Every sixth section was stained with Toluidine blue, and infarct volume was estimated based on Cavalieri’s principle for volume estimation as previously described (Gregersen et al., 2000; Lambertsen et al., 2009). The rostrocaudal distribution of the infarct was estimated 3,600 μm anterior of the anterior commissure to 3,600 μm posterior of the anterior commissure as previously described (Clausen et al., 2016a; Madsen et al., 2016).

### Real-Time Polymerase Chain Reaction (RT-PCR)

One series of brain tissue was used for RT-PCR. The RNA was extracted using the TRIzol (Invitrogen) method as previously described (Meldgaard et al., 2006). Purity of RNA was controlled spectrophotometrically with 260/280 and 230/260 values using NanoDrop (Thermo Fisher Scientific). Degradation of RNA was measured by RNA integrity factor (RIN) using Agilent 2100 Bioanalyzer (Agilent Genomics). Samples were used for PCR if they had a RIN factor of 7.6 or above. RNA was reverse transcribed into complementary DNA (cDNA) according to the High-Capacity cDNA Reverse Transcription kit protocol (Applied Biosystems). PCR was performed according to the Maxima SYBR Green/ROX qPCR Master Mix (2X) (Thermo Fisher Scientific) protocol. Primer sequences used for RT-PCR (Table 1) were designed to target exon-exon junctions whenever possible. Primers were ordered from TAG Copenhagen. Values were normalized to hypoxanthine phosphoribosyltransferase 1 (HPRT1) mRNA, and relative values were calculated relative to a calibrator pool of unmanipulated mice as previously described (Meldgaard et al., 2006).

**TABLE 1:** Primer sequences for RT-PCR.

**Gene**	**Primer sequence**	**Product length**
CD11b	F: 5′ cggaaagtagtgagagaactgtttc 3′	114 bp
	R: 5′ cttataatccaagggatcaccgaattt 3′	
CX3CR1	F: 5′ cctgcctctgagaaatggag 3′	332 bp
	R: 5′ atctctccagcccctgaaat 3′	
	F: 5′ cagcatcgaccggtacctt 3′	65 bp
	R: 5′ gctgcactgtccggttgtt 3′	
Arg1	Mm00475988_m1 from Life technologies	65 bp
CCL2	F: 5′ ccccactcacctgctgctac 3′	86 bp
	R: 5′ cctgctgctggtgattctctt 3′	
CXCL10	F: 5′ gccgtcattttctgcctcatcct 3′	113 bp
	R: 5′ ctcattctcactggcccgtcatc 3′	
CXCL1	F: 5′ catggctgggattcacctcaag 3′	113 bp
	R: 5′ ggcaagcctcgcgaccattct 3′	
TREM2	F: 5′ cagccctgtcccaagccctcaac 3′	134 bp
	R: 5′ ctcctcacccagctgccgacacc 3′	
P2RY12	F: 5′ tctttgctgggctcatcacgaa 3′	167 bp
	R: 5′ aggcccggctcccagtttag 3′	
iNOS	F: 5′ ggacagcacagaatgttccagaa 3′	104 bp
	R: 5′ caaaatctctccactgccccag 3′	
IL-1β	F: 5′ tgtaatgaaagacggcacac 3′	68 bp
	R: 5′ tcttctttgggtattgcttgg 3′	
TNFR1	F: 5′ gcccgaagtctactccatcatttg 3′	91 bp
	R: 5′ ggctggggagggggctggagttag 3′	
TNFR2	F: 5′ gcccagccaaactccaagcatc 3′	133 bp
	R: 5′ tcctaacatcagcagacccagtg 3′	
HPRT1	F′: 5′-aagcagtacagccccaaaatg-3′	
	R′: 5′-aaatccaacaaagtctggcctgta-3′	

**F, forward; R, reverse.**

### Cytokine and Receptor Protein Expression and Drug Concentration Analysis

One series of brain tissue was lysed in Complete Mesoscale buffer according to the manufacturer’s protocol (Mesoscale Discovery, Rockville, MD, United States). Protein concentrations were determined according to the protocol from Micro BCA protein assay kit (Thermo Fischer Scientific). Cytokine and chemokine concentrations were estimated using an MSD Mouse Pro-Inflammatory V-Plex Plus Kit (Mesoscale Discovery) and TNF receptor concentrations using the Mouse TNF-RI and TNFR-II Ultra-Sensitive Kits (Mesoscale Discovery). XPro1595 levels were measured in ischemic brain tissue lysates using a human TNF V-Plex immunoassay (Mesoscale Discovery) as previously described (Karamita et al., 2017). The standard in the kit was replaced with XPro1595, which was diluted in the kit diluent number 2. Etanercept levels were measured using a human TNFRII Ultra-Sensitive immunoassay (Mesoscale Discovery) (Karamita et al., 2017). The standard in the kit was replaced with etanercept diluted in the kit diluent number 2. All kits were read on a SECTOR Imager 6000 plate reader (Mesoscale Discovery) according to the manufacturer’s instructions. All samples were run in duplex, and coefficient of variation (CV) values below 25% were accepted.

### Characterization of Cells From Mice With Genetic Ablation of SolTNF

#### Cell Purification for Proteomics

Microglia and neurons were purified from cortices from adult (8 weeks) tmTNF^Δ/Δ^ and tmTNF^wt/wt^ mice by MACS affinity-based cell sorting (Miltenyi Biotech, Bergisch-Gladbach, Germany) as previously described (Clausen et al., 2016b). Mice were sacrificed by cervical dislocation, cortices dissociated with Neuronal dissociation papain kit (P) (Miltenyi Biotech) and microglia and neurons purified according to the manufacturer’s protocol. The cell suspensions were incubated with monoclonal CD11b beads (Miltenyi Biotech) and applied to LS magnetic separation columns (Miltenyi Biotech) attached to a magnetic field for positive selection of microglia. The flow through was further incubated with Neuron Isolation Kit (Miltenyi Biotech) and applied to LD magnetic separation columns (Miltenyi Biotech) for negative selection of neurons. The cell pellets were stored at −80°C until preparation for mass spectrometry.

#### Sample Preparation/Digestion for Mass Spectrometry

Microglia and neurons were prepared using a filter-aided sample preparation protein digestion protocol essentially according to Kruse Meyer et al. (2015). Briefly, samples were lysed in cold lysis buffer (5% sodium deoxycholate, 50 mM triethylammonium bicarbonate, pH 8.5) and homogenized by bead beating (Bullet Blender Gold (NextAdvance, United States); 0.9–2.0 mm steel bead blend; Setting 10; 2 × 5 min; +4°C) and heat-denaturized at 95°C for 5 min. Protein concentrations were estimated using a NanoDrop 1000 UV-Vis spectrophotometer (Thermo Fisher Scientific) using bovine serum albumin as reference standard. Protein lysate was transferred to a 10 kDa molecular weight cutoff spin-filter (Millipore, Billerica, MA, United States) and protein disulfide bonds were reduced with 10 mM tris(2-carboxyethyl)phosphine (Thermo Fisher Scientific) for 30 min. Afterward, cysteine residues were blocked with 50 mM chloroacetamide (Sigma-Aldrich, St. Louis, MO, United States) for 30 min in the dark. Protein digestion was performed with sequencing grade modified trypsin (Promega, Madison, WI, United States) at an enzyme to substrate ratio of 1:50 (w/w) for 16 h at 37°C. The peptide material was eluted from the spin-filter, acidified with trifluoroacetic acid to a final concentration of 1% and purified by phase inversions with ethyl acetate 1:2 (v/v). The peptide rich aqueous phase was dried down in a vacuum centrifuge.

#### UPLC-MS/MS Sample Analysis

Ultra-performance liquid chromatography-tandem mass spectrometry (UPLC-MS/MS) analysis was performed using an UltiMate 3000 UPLC system (Thermo Fisher Scientific) coupled online to a Q Exactive HF mass spectrometer (Thermo Fisher Scientific). Peptide material was separated on a 75 cm C18 Acclaim PepMap100 analytical column (Thermo Fisher Scientific) with 96% solvent A (0.1% FA) and 4% solvent B (0.1% FA in acetonitrile), which was increased to 30% solvent B on a 60 min ramp gradient at a constant flow rate of 250 nL/min. Eluting peptides were introduced directly into the mass spectrometer by a picotip emitter for electrospray ionization (New Objective, Woburn, MA, United States).

The mass spectrometer was operated in positive mode using a data-dependent TOP12 acquisition method with the following settings: mass range m/z 400–1200; isolation window m/z 1.6; NCE 27; charge state exclusion: unassigned, 1, >6; peptide match preferred; dynamic exclusion 30 s.

#### Protein Quantification and Filtering

The .RAW files were investigated by Progenesis QI for Proteomics to access the chromatographic quality. The raw data were searched with two complementary label free quantitative approaches: MaxQuant (v1.5.6.0) against the UniProt Mouse reference proteome database with isoforms (UP000000589, November 2016). Standard settings were employed in MaxQuant, including max two tryptic missed cleavages, and a first search with 20 ppm mass tolerance followed by data calibration and a main search with 4.5 ppm mass tolerance. The match between runs feature was enabled to allow the transfer of identified precursors between different runs, based on accurate mass and retention time. The following peptide modifications were found to be abundant with the applied protocol and were included in the search: carbamidomethylated cysteine residues (fixed), acetylation of the N-terminal of proteins (variable), oxidation of methionine (variable). Identified proteins and peptide spectral matches were filtered to <1% false discovery rate (FDR) using a forward/reverse database search strategy in MaxQuant, and proteins tagged as common contaminants were removed. Relative protein quantities were calculated by summing the unique peptide peak areas of each protein in MaxQuant using the LFQ (label-free quantitation) feature. Additional filtering steps were employed in Perseus (v1.5.6.0): (1) The quantitation of any protein was required to be based on at least two unique peptides. (2) Unique peptides were required to be quantifiable in at least three of the tmTNF^wt/wt^ or tmTNF^Δ/Δ^ samples. (3) Pearson’s correlation coefficients (R) between the technical repeats should be greater than 0.95, based on log2 transformed protein LFQ values.

#### Data Processing

All abundances were log2-transformed and proteins with less than two unique peptides and (>50% missing values) filtered out. Proteins with a statistically significant mean abundance difference between the tmTNF^wt/wt^ and tmTNF^Δ/Δ^ samples were identified by independent two-sample t-tests in Perseus, with permutation-based truncation using standard parameters (s0 = 0.1, 250 randomizations) to correct for multiple hypothesis testing. In this way, the p-value requirement was adjusted to expect <5% false positives among the statistically significant findings.

The MS data has been deposited to the ProteomeXchange Consortium^1^ via the PRIDE partner repository with dataset identifiers PXD014440. Protein-protein interaction analysis was performed by STRING^2^.

### Phagocytosis Assay

In order to test phagocytic activity of primary microglia derived from tmTNF^wt/wt^ and tmTNF^Δ/Δ^ mice, the microglia were plated, activated and incubated with fluorescent beads. As a measure of phagocytic activity, the number of engulfed beads was counted and morphological changes estimated as previously described (Al-Ali et al., 2017; Gao et al., 2017). Microglial cultures were prepared using brain tissue from tmTNF^Δ/Δ^ (*n* = 3) and tmTNF^wt/wt^ (*n* = 6) pups at post-natal age 5–7. The meninges and blood vessels were removed, and brains collected in ice-cold HBSS without Ca^2+^ and Mg^2+^ (Thermo Fisher Scientific) under sterile conditions. Cortices were processed using Neural Tissue Dissociation Kit (P) (Miltenyi Biotech) according to the manufacturer’s protocol. Cell suspensions were filtered through 70 μm cell strainers (BD Biosciences, San Jose, CA, United States) and washed with HBSS with Ca^2+^ and Mg^2+^ (Thermo Fisher Scientific). Cell suspensions were centrifuged, and the pellet used for MACS sorting. Microglia were purified by MACS sorting using magnetic CD11b^+^ beads on LS columns (Miltenyi Biotech) and cultured in culture medium A (76 % Dulbecco’s modified Eagle’s medium (DMEM) (Thermo Fisher Scientific), 20% FBS (Thermo Fisher Scientific), 1% minimum essential media (MEM) (Thermo Fisher Scientific), 1% penicillin streptomycin (Thermo Fisher Scientific), 1% Glutamax^TM^ (Gibco, Thermo Fisher Scientific), 1% pyruvate (Thermo Fisher Scientific)) on poly-L-ornithine-coated plates in a humified CO_2_ incubator at 37°C. After 3 days half of the media was changed to culture medium B (86% DMEM, 10% FBS, 1% MEM, 1% penicillin streptomycin, 1% Glutamax^TM^, 1% pyruvate). Four days after the media change microglia were activated by adding 100 ng/mL LPS (Invitrogen, Thermo Fisher Scientific) for 24 h. After activation, FluoSpheres^TM^ Carboxylate-Modified Microspheres (1.0 μm, 505/515, yellow-green fluorescent) (Thermo Fisher Scientific) were added onto the cultured cells. After 2 h phagocytosis was terminated by adding cold culture medium B. Cells were fixed with 4% paraformaldehyde, blocked in 5% normal goat serum in tris-buffered saline and incubated overnight in anti-Iba1 antibody (1:500, Fujifilm Wako Pure Chemical Corporation, Japan). The next day, cells were stained using alexa 594-conjugated anti-rabbit antibody (1:750, Alexa594 Molecular Probes) (Thermo Fisher Scientific) and 4′,6-diamidino-2-phenylindol (DAPI) (Thermo Fisher Scientific). Pictures were taken using a FV1000 (Olympus) confocal microscope from nine randomly chosen positions per coverslip. Analysis of phagocytosis measured as engulfed beads/cell and cell morphology measured as cell area, membrane irregularity and perimeter length was done by automated counting software Puntomorph^3^ (Al-Ali et al., 2017).

### Statistical Analysis

Data are presented as mean ± SEM. Statistical comparison between the three groups (saline, Xpro1595, and etanercept) was assessed by one-way ANOVA followed by Tukey’s *post hoc* test (GraphPad Prism Software Inc., San Diego, CA, United States). Comparison of the rostrocaudal distribution of the infarct and comparison of phagocytosis in tmTNF^Δ/Δ^ and tmTNF^wt/wt^ microglia were tested by two-way ANOVA followed by Tukey’s *post hoc* test. Statistical significance of the asymmetry of the paws in the grip strength test and the Hargreaves nociception test was tested by Student’s paired *t*-test. Normal distribution of variables was tested using both Brown-Forsythe and Bartlett’s tests. Significance was set at *p* ≤ 0.05.

## Results

### Topical Delivery of XPro1595 Decreases Infarct Volume 1 and 3 Days After pMCAO

**FIGURE 1:**
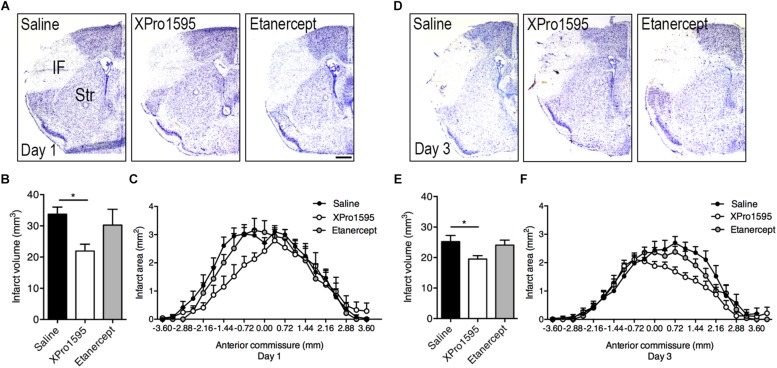
Infarct volume and rostrocaudal distribution after topical administration of TNF inhibitors. **(A)** Toluidine blue-stained brain sections of mice treated topically with saline, XPro1595, or etanercept and allowed to survive 1 day after pMCAO. Scalebar 500 μm. **(B)** Infarct volume estimation 1 day after pMCAO. N(saline) = 11; n(XPro1595) = 6; n(etanercept) = 4. **(C)** Rostrocaudal distribution of the infarct 1 day after pMCAO. **(D)** Toluidine blue-stained brain sections of mice treated topically with saline, XPro1595, or etanercept and allowed to survive 3 days after pMCAO. **(E)** Infarct volume estimation 3 days after pMCAO. N(saline) = 12; n(XPro1595) = 15; n(etanercept) = 17. **(F)** Rostrocaudal distribution of the infarct 3 days after pMCAO. ^*^p ≤ 0.05, one-way ANOVA followed by Tukey’s *post hoc* test.

Infarct volumes were estimated in Toluidine blue-stained brain sections from mice treated topically or i.c.v. with saline, XPro1595, or etanercept and allowed to survive 1 or 3 days after pMCAO (Figure 1 and Supplementary Figure 1A). At day 1, infarct volumes were significantly reduced in mice treated topically with XPro1595 compared to mice treated topically with saline (Figure 1B). Etanercept treatment showed no effect. The rostrocaudal distribution of the infarcts was comparable between groups (Figure 1C). At day 3, infarct volumes were also significantly reduced in mice treated topically with XPro1595 (Figure 1E). Again, the rostrocaudal distribution of the infarct was comparable between groups (Figure 1F).

Intracerebroventricular administration of XPro1595 or etanercept was not effective in decreasing infarct size (Supplementary Figure 1A). In support of this, we observed no difference in functional outcomes as assessed by the Hargreaves thermal hyperalgesia (Supplementary Figure 1B) or grip strength tests (Supplementary Figures 1C,D), except for a total weaker grip strength on day 3 compared to baseline in mice treated with etanercept (Supplementary Figure 1D).

### XPro1595 and Etanercept Can Be Detected in Brain Ischemic Tissue

**FIGURE 2:**
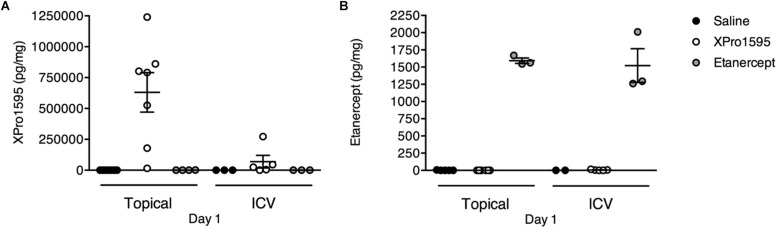
Detection of TNF inhibitors in ischemic brain tissue 24 h after pMCAO. **(A)** Concentration of XPro1595 in brain tissue after topical and i.c.v. administration. *N* = 4–7/group (excluded due to too high CV values: saline pump, *n* = 4; saline i.c.v., *n* = 2). **(B)** Concentration of etanercept in brain tissue after topical and i.c.v. administration. *N* = 2–6/group (excluded due to too high CV values: saline i.c.v., *n* = 2; XPro1595 i.c.v., *n* = 1).

To validate the presence of XPro1595 and etanercept and estimate their concentrations in the ischemic brain following topical and i.c.v. delivery, we measured the concentration of XPro1595 and etanercept 1 day after pMCAO (Figures 2A,B). We found topical administration to be an efficient method to deliver a high concentration of XPro1595 to neural tissue (Figure 2A). After topical administration, the concentration of XPro1595 in brain tissue 1 day after pMCAO was 630,300 ± 160,000 pg/mg. The concentration of XPro1595 was lower after i.c.v. administration, with a mean of 69,400 ± 51,300 pg/mg (Figure 2A). This concentration in the neural tissue is, however, sufficient to neutralize solTNF and to promote neuroprotection, based on previous publications in an animal model of Alzheimer’s disease (MacPherson et al., 2017).

Etanercept was detected in the brain tissue after both delivery routes (Figure 2B). The concentration of etanercept in brain tissue 1 day after pMCAO was 1,600 pg/mg after topical administration and 1,500 ± 200 pg/mg after i.c.v. administration. This demonstrated that both XPro1595 and etanercept successfully accessed the ischemic brain tissue.

### Topical Treatment With XPro1595 Does Not Affect Microglial and Leukocyte Cell Populations After pMCAO

**FIGURE 3:**
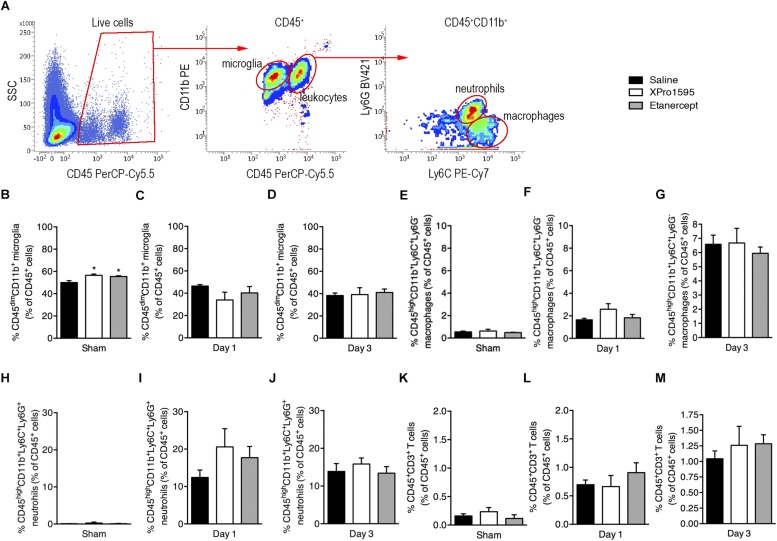
Flow cytometric analysis of ipsilateral hemispheres in sham and pMCAO mice after topical saline, XPro1595, or etanercept treatment**. (A)** Gating strategy for CD45^+^ cells, CD45^dim^CD11b^+^ microglia, CD45^high^CD11b^+^ leukocytes, CD45^high^CD11b^+^Ly6C^+^Ly6G^–^ macrophages, and CD45^high^CD11b^+^Ly6C^+^Ly6G^+^ granulocytes. **(B–D)** Changes in CD45^dim^CD11b^+^ microglia presented as % of CD45^+^ cells 3 days after sham surgery or 1 or 3 days after pMCAO. **(E–G)** Changes in CD45^high^CD11b^+^Ly6C^+^Ly6G^–^ macrophages presented as % of CD45^+^ cells after sham surgery or 1 or 3 days after pMCAO. **(H–J)** Changes in CD45^high^CD11b^+^Ly6C^+^Ly6G^+^ granulocytes presented as % of CD45^+^ cells after sham surgery or 1 or 3 days after pMCAO. **(K–M)** Changes in CD3^+^ T cells presented as % of CD45^+^ cells after sham surgery or 1 or 3 days after pMCAO. N(sham) = 3–5/group; n(day 1) = 4–5/group; n(day 3) = 5/group; ^*^p ≤ 0.05; one-way ANOVA followed by Tukey’s *post hoc* test.

To investigate potential changes in microglial and leukocyte populations in mice subjected to pMCAO and treated topically with saline, XPro1595 or etanercept, we performed flow cytometry and gated for CD45^dim^CD11b^+^ microglia, CD45^dim^CD11b^+^Ly6C^+^Ly6G^–^ macrophages, CD45^dim^CD11b^+^Ly6C^+^Ly6G^+^ granulocytes (Figure 3A), and CD45^+^CD3^+^ T cells.

We found that compared to saline, both Xpro1595 and etanercept treatment increased the percentage of microglia in the ipsilateral cortex 3 days after sham surgery (Figure 3B), whereas no difference was observed between groups at day 1 (Figure 3C) or day 3 (Figure 3D) after pMCAO. In all groups of mice 3 days after pMCAO, the microglial population constituted ∼80% of all live CD45^+^ cells in the contralateral cortex, with only ∼60% in sham. By 1 day after pMCAO the microglial population constituted ∼60% of all live CD45^+^ cells (Supplementary Figures 2A–C). The percentage of infiltrating macrophages (Figures 3E–G), granulocytes (Figures 3H–J), and T cells (Figures 3K–M) did not differ between groups at any time point investigated, although the percentages of cells increased over time in the ipsilateral cortex (Figures 3E–M) and remained relatively constant in the contralateral cortex (Supplementary Figures 2D–L).

Since microglia and macrophages upregulate their expression of CD11b and CD45 after ischemia (Ito et al., 2001; Ponomarev et al., 2005; Clausen et al., 2008; Morrison and Filosa, 2013), their activation status was evaluated based on the MFI of these cell surface markers. MFIs of CD11b and CD45 were comparable between treatment groups 3 days after sham surgery and 1 and 3 days after pMCAO (Supplementary Figures 3A–L). However, both microglia and leukocytes seemed to downregulate their expression of CD11b and upregulate their expression of CD45 3 days after pMCAO, which was not the case in sham mice and 1 day after pMCAO (Supplementary Figures 3A–L, please compare C to A and B; I to G and H; F to D and E; and L to J and K).

### XPro1595 Affects Microglial Activation

**FIGURE 4:**
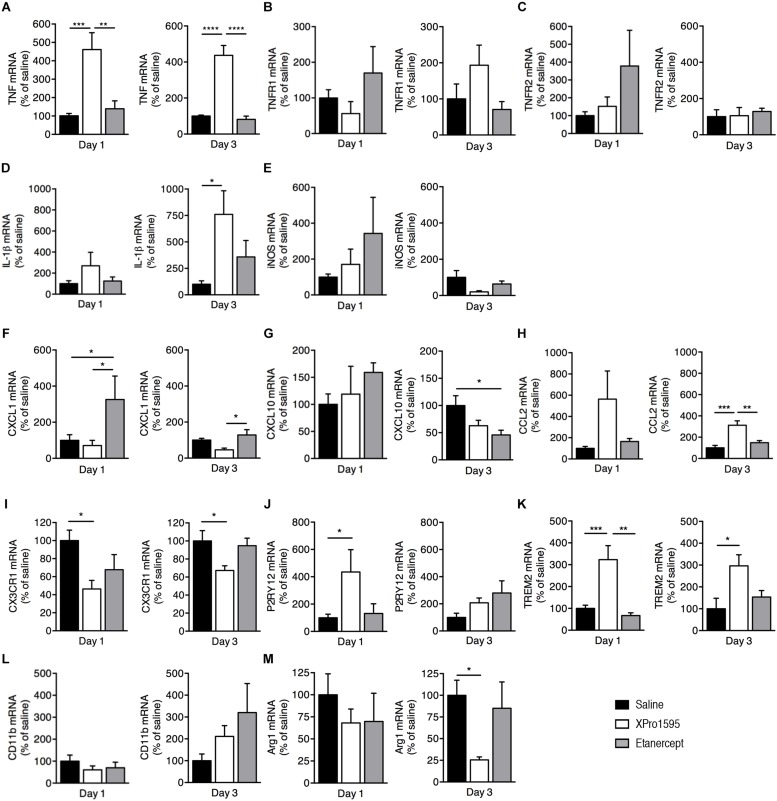
Inflammatory gene profile 1 and 3 days after pMCAO in mice treated topically with saline, XPro1595, or etanercept. mRNA expression of cytokines, pro-inflammatory molecules, chemokines, and microglial markers presented as % of gene expression in saline-treated animals **(A–M). (A)** Expression of *Tnf* mRNA. **(B)** Expression of *Tnfrsf1a* (TNFR1) mRNA. **(C)** Expression of *Tnfrsf1b* (TNFR2) mRNA. **(D)** Expression of *Il1β* mRNA. **(E)** Expression of *Nos2* (iNOS) mRNA. **(F)** Expression of *Cxcl1* mRNA. **(G)** Expression of *Cxcl10* mRNA. **(H)** Expression of *Ccl2* mRNA. **(I)** Expression of *Cx3cr1* mRNA. **(J)** Expression of *P2ry12* mRNA. **(K)** Expression of *Trem2* mRNA. **(L)** Expression of *CD11b* mRNA. **(M)** Expression of *Arg1* mRNA. N(day 1) = 4–11/group, n(day 3) = 5–6/group; ^*^p ≤ 0.05, ^∗∗^*p* ≤ 0.01, ^∗∗∗^*p* ≤ 0.001, ^****^p ≤ 0.0001; one-way ANOVA followed by Tukey’s *post hoc* test.

We next investigated changes in mRNA expression of selected microglial/macrophage and inflammatory markers in brain tissue derived from mice subjected to pMCAO and treated topically (Figure 4) or i.c.v. (Supplementary Figure 4) with saline, XPro1595, or etanercept and allowed to survive 1 or 3 days after pMCAO.

We found *Tnf* mRNA expression to be significantly upregulated 1 and 3 days after pMCAO in mice treated topically with XPro1595 when compared to saline- and etanercept-treated mice (Figure 4A). *Tnfrsf1a* (TNFR1, Figure 4B) and *Tnfrsf1b* (TNFR2, Figure 4C) mRNA expression was comparable between treatment groups at both days 1 and 3 after pMCAO. At 3 days, *Il-1beta* mRNA levels were significantly upregulated in mice treated topically with XPro1595 compared to saline- and etanercept-treated mice (Figure 4D), whereas no significant changes in *Nos2* (iNOS) mRNA expression was observed between groups, although XPro1595 tended to downregulate the expression at 3 days (*p* = 0.08; Figure 4E).

To study whether TNF inhibition using XPro1595 or etanercept affects chemokine gene expression, we measured the mRNA levels of *Cxcl1*, *Cxcl10*, and *Ccl2* at 1 and 3 days after pMCAO. Topical etanercept treatment significantly increased *Cxcl1* mRNA compared to saline and XPro1595 at day 1 and compared to XPro1595 at day 3 after pMCAO (Figure 4F). In contrast, *Cxcl10* mRNA levels decreased at day 3 in mice treated topically with etanercept compared to saline (Figure 4G), and *Ccl2* mRNA levels significantly increased at day 3 in XPro1595-treated mice compared to both saline- and etanercept-treated mice (Figure 4H).

We further measured mRNA expression levels of microglial/macrophage markers *Cx3cr1*, *P2ry12*, *Trem2*, *Cd11b*, and *Arg1*. We found that at days 1 and 3 after pMCAO, topical XPro1595 treatment decreased *Cx3cr1* mRNA expression compared to saline-treated mice (Figure 4I). In contrast, *P2ry12* mRNA expression was significantly increased at 1 day after pMCAO in topically XPro1595-treated mice compared to saline-treated mice (Figure 4J), as well as *Trem2* mRNA expression at 1 and 3 days after pMCAO (Figure 4K). *Cd11b* and *Arg1* mRNA levels were comparable between groups except for a significant decrease in *Arg1* mRNA levels in XPro1595-treated mice at day 3 (Figures 4L,M).

Even though i.c.v. administration of TNF inhibitors had no effect on infarct volume or functional outcome, we also investigated potential changes in mRNA expression after i.c.v. injection of XPro1595 or etanercept (Supplementary Figure 4). We observed no change in *Tnf*, *Tnfrsf1a*, *Tnfrsf1b*, *Il-1beta*, *Nos2*, *Cxcl1*, *Cx3cr1*, or *CD11b* mRNA expression between groups. Etanercept significantly increased *Cxcl10* mRNA levels compared to saline at day 1 and compared to both XPro1595 and saline 3 days after pMCAO (Supplementary Figure 4). Also, *P2ry12* mRNA levels increased significantly at day 1 in etanercept-treated mice compared to saline- and Xpro1595-treated mice, and *Ccl2* and *Trem2* mRNA levels increased significantly at day 3, while *Arg1* decreased significantly.

### Cytokine, Chemokine, and TNF Receptor Expression in Brain Tissue 1 and 3 Days After TNF Inhibitor Treatment

**FIGURE 5:**
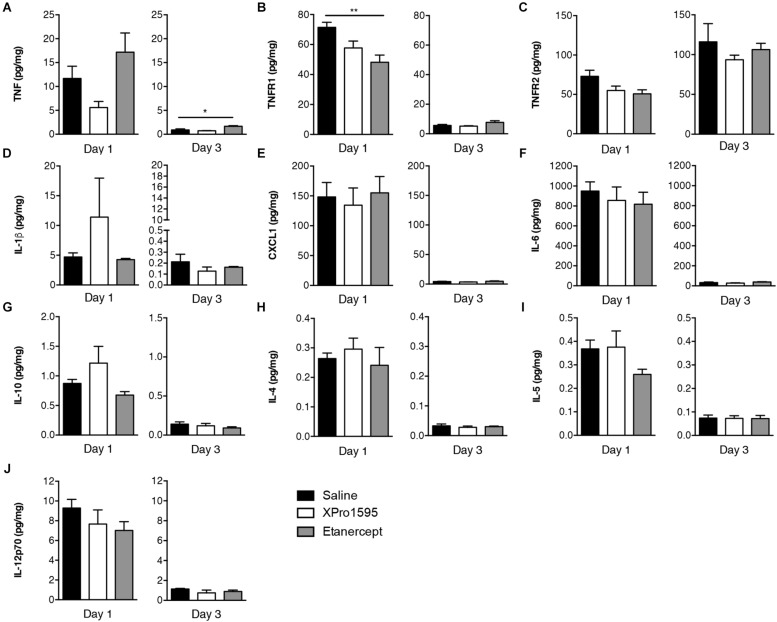
Cytokine and TNF receptor protein expression in brain tissue 1 and 3 days after pMCAO in mice treated topically with saline, XPro1595, or etanercept. **(A)** Expression of TNF (*n* = 4–11/group). **(B)** Expression of TNFR1 (*n* = 4–11/group). **(C)** Expression of TNFR2 (*n* = 4–10/group). **(D)** Expression of IL-1β (*n* = 3–11/group). **(E)** Expression of CXCL1 (*n* = 3–11/group). **(F)** Expression of IL-6 (*n* = 3–9/group). **(G)** Expression of IL-10 (*n* = 3–10/group). **(H)** Expression of IL-4 (*n* = 2–11/group). **(I)** Expression of IL-5 (*n* = 4–10/group). **(J)** Expression of IL-12p70 (*n* = 4–11/group). ^∗∗^*p* ≤ 0.01; one-way ANOVA with Tukey’s *post hoc* test.

To study whether topical (Figure 5) or i.c.v. (Supplementary Figure 5) XPro1595 and etanercept treatment after pMCAO affected cytokines, TNF receptor and/or chemokine protein levels, we measured TNF, TNFR1, TNFR2, IL-1β, CXCL1, IL-6, IL-10, IL-4, IL-5, and IL-12p70 levels in brain tissue 1 day after pMCAO, when neuroinflammation is maximal (Lambertsen et al., 2012), in addition to 3 days after pMCAO using multiplex ELISA.

We found that despite increased *Tnf* mRNA expression after topical XPro1595 treatment, the protein expression tended to be downregulated in XPro1595-treated mice (*p* = 0.06, Figure 5A), whereas TNF levels in etanercept-treated mice were comparable to saline-treated mice 1 day after pMCAO. At 3 days, TNF levels had increased significantly in etanercept-treated mice compared to saline-treated mice. Compared to topical saline treatment, etanercept treatment downregulated TNFR1 levels at day 1, which also showed tendency to be downregulated after XPro1595 treatment (*p* = 0.08), whereas TNFR1 levels were comparable at 3 days (Figure 5B). Topical treatment had no effect on expression of TNFR2, IL-1β, Cxcl1, IL-6, IL-10, IL-4, IL-5, or IL-12p70 (Figures 5C–J).

Intracerebroventricular administration of TNF inhibitors showed no effect on the TNF or TNFR1 protein expression (Supplementary Figures 5A,B) although TNFR2 was downregulated after etanercept-treatment compared to saline treatment 1 day after pMCAO (Supplementary Figure 5C). Treatment with XPro1595 showed a tendency toward reducing TNFR2 levels at day 1 after pMCAO (*p* = 0.07) (Supplementary Figure 5C). At 1 day after pMCAO, etanercept treatment significantly increased IL-1β levels compared to saline (Supplementary Figure 5D), but no effect was observed on CXCL1, IL-6, IL-10, IL-4, IL-5, or IL-12p70 levels (Supplementary Figures 5E–J).

### tmTNF Microglia Have an Upregulated Expression of Phagocytosis-Related Proteins

**FIGURE 6:**
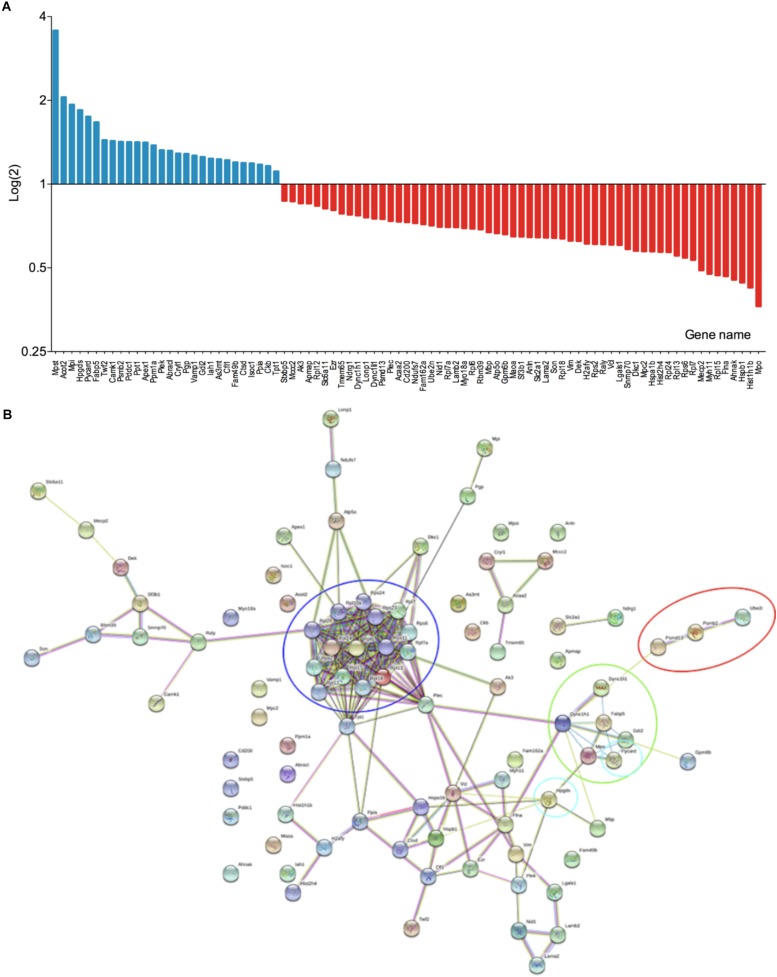
Proteome analysis of primary microglia from tmTNF^wt/wt^ and tmTNF^Δ/Δ^ mice. **(A)** Differentially expressed proteins in tmTNF^wt/wt^ and tmTNF^Δ/Δ^ microglia (*n* = 4/group). **(B)** String analysis of differentially expressed proteins in tmTNF^wt/wt^ and tmTNF^Δ/Δ^ microglia. Red circle, FCERI-mediated NF-kappa B activation; blue circle, ribosomal proteins; green circle, neutrophil degranulation; turquoise circles, proteins related to phagocytosis.

In order to investigate the hypothesis that ablation of solTNF improves microglial phagocytosis, we investigated differentially expressed proteins in microglia derived from tmTNF^Δ/Δ^ and tmTNF^wt/wt^ mice using mass spectrometry. In total, 1,564 proteins with two or more unique peptides were quantified by label free quantification in microglia cells (Supplementary Table 1). Of these, 87 proteins were differentially regulated between tmTNF^wt/wt^ and tmTNF^Δ/Δ^ mice (Figure 6A). Several of the upregulated proteins, 3-mercaptopyruvate sulfurtransferase (3-MPST), hematopoietic prostaglandin D synthase (HPGDS) and apoptosis-associated speck-like protein containing a CARD (PYCARD/ASC) are neuroprotective (Liu et al., 2009; Chen et al., 2015; Kimura et al., 2017; Shan et al., 2017) or related to regulation of phagocytosis (Rajakariar et al., 2007; Ippagunta et al., 2011). 3-MPST has previously been shown to be upregulated in activated microglia treated with a neuroprotective compound (Cobourne-Duval et al., 2018). tmTNF expressing microglia had an increased expression of HPGDS, which is known to be expressed in activated microglia in the core of the infarct after ischemic stroke and to enhance their differentiation into a phagocytic phenotype (Mohri et al., 2003; Liu et al., 2007). PYCARD/ASC supports the function of a normal immune system (Ippagunta et al., 2011). Among the downregulated proteins was MPO. Interestingly, upregulation of myeloperoxidase (MPO) expression is linked to pathological phagocytosis of myelin leading to demyelination in patients with multiple sclerosis (Minohara et al., 2006; Gray et al., 2008). Moreover, the expression of Methyl-CpG-binding protein 2 (MECP2) (Cronk et al., 2015) was downregulated in tmTNF expressing microglia. The absence of microglial MECP2 has shown to increase *Tnf* mRNA expression (Cronk et al., 2015). Interestingly, tmTNF expressing microglia had downregulated expression of Histone H1.5, which promotes microglial proinflammatory functions (Gilthorpe et al., 2013), Heat shock protein 27, which is neuroprotective in ischemic brain (Stetler et al., 2012) and Heat shock 70 kDa protein 1, which functions as a chaperone (Stetler et al., 2010) supporting earlier studies showing solTNF to be needed for optimal control of inflammatory response (Ruuls et al., 2001; Musicki et al., 2006; Dambuza et al., 2011; DeBerge et al., 2014). Downregulation of Human growth and transformation-dependent protein (HGTD-P), a protein which is proapoptotic in ischemia (Qu et al., 2009), and downregulation of Vimentin, which has been shown to mediate microglial activation (Jiang et al., 2012), supports the beneficial function of tmTNF. Two other histones, Histone H4 and Core histone macro-H2A.1 were downregulated in tmTNF expressing microglia. String analysis of the differentially regulated proteins in microglia showed clustering of the aforementioned immune system-related proteins Histone H1.5, Heat shock protein 27, Methyl-CpG-binding protein 2, Heat shock 70 kDa protein, Core histone macro-H2A.1, Vimentin, HPGDS, and Histone H4 (Figure 6B).

### Microglia Expressing Only tmTNF Display Improved Phagocytic Properties

**FIGURE 7:**
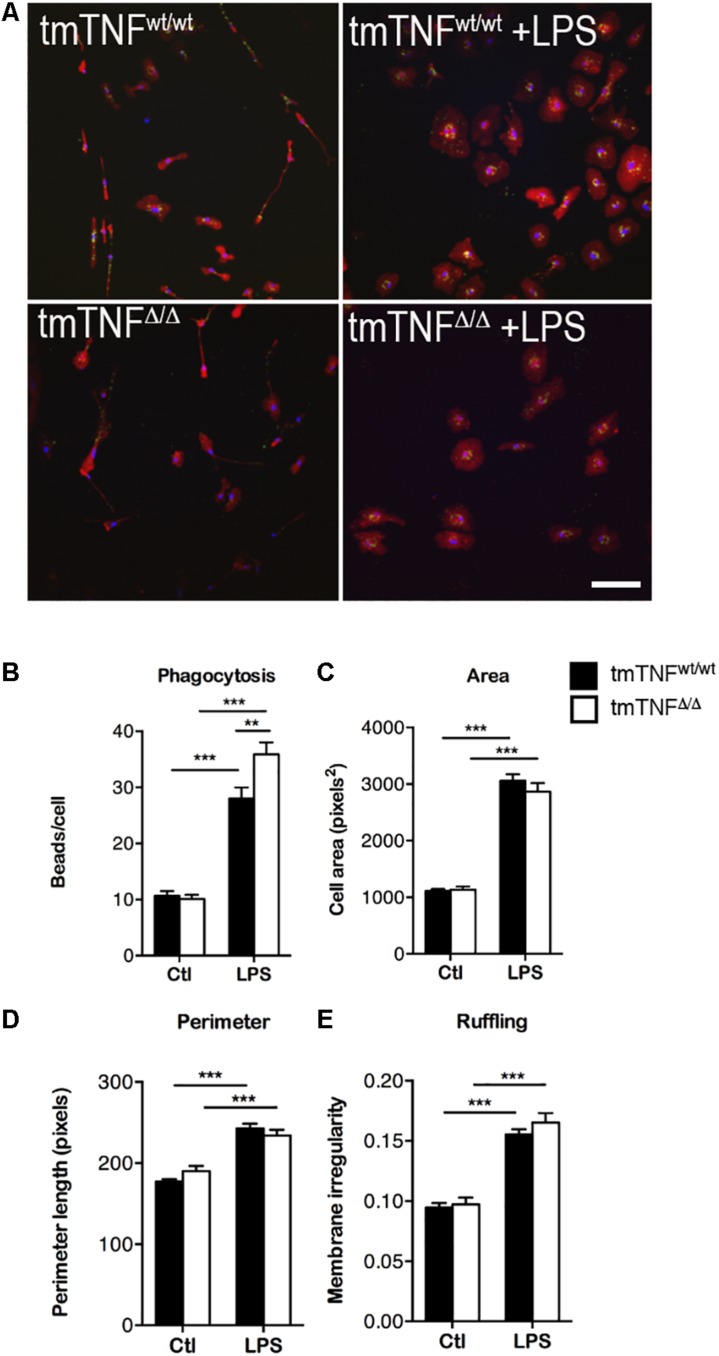
Microglia from tmTNF^Δ/Δ^ mice have increased phagocytic activity after LPS activation. **(A)** Primary microglia cultures stained for Iba1 (red) and DAPI (blue) demonstrating phagocytosis of FluoSpheres Carboxylate-Modified Microspheres (green). Staining of tmTNF^wt/wt^ microglia (upper left), tmTNF^wt/wt^ + LPS (upper right), tmTNF^Δ/Δ^ (lower left), tmTNF^Δ/Δ^ + LPS (lower right). Scalebar = 100μm. **(B)** Engulfed beads/cell in tmTNF^wt/wt^ and tmTNF^Δ/Δ^ microglia. N(tmTNF^wt/wt^) = 426 cells/25 FOV, n(tmTNF^wt/wt^ + LPS) = 282 cells/17 FOV, n(tmTNF^Δ/Δ^) = 169 cells/20 FOV, n(tmTNF^Δ/Δ^ + LPS) = 137 cells/20 FOV. [Two-way ANOVA: treatment ^∗∗∗^*p* < 0.001, *F*_(1, 78)_ = 6.03; genotype ^*^p < 0.05, *F*_(1, 78)_ = 210.1; interaction ^∗∗^*p* < 0.01 *F*_(1, 78)_ = 8.05]. **(C–E)** Morphological features of microglia before and after LPS activation. **(C)** Area. [Two-way ANOVA: treatment ^∗∗∗^*p* < 0.001, *F*_(1, 78)_ = 373.3]. **(D)** Perimeter. [Two-way ANOVA: treatment ^∗∗∗^*p* < 0.001, *F*_(1, 78)_ = 96.08]. **(E)** Ruffling. [Two-way ANOVA: treatment ^∗∗∗^*p* < 0.001, *F*_(1, 78)_ = 132.5]. For C-E, N(tmTNF^wt/wt^) = 426 cells/25 FOV, n(tmTNF^wt/wt^ + LPS) = 282 cells/17 FOV, n(tmTNF^Δ/Δ^) = 169 cells/20 FOV, n(tmTNF^Δ/Δ^ + LPS) = 137 cells/20 FOV. LPS, lipopolysaccharide; FOV, field of view.

Due to the upregulated expression of phagocytosis-related proteins in microglia expressing only tmTNF, we next investigated the phagocytic properties and morphological changes 1 day after LPS stimulation in primary microglia derived from 5 to 7 days old tmTNF^Δ/Δ^ and tmTNF^wt/wt^ pups (Figure 7A). Phagocytosing microglia have a neuroprotective role in clearing apoptotic cells and debris after stroke (Neumann et al., 2009; Sierra et al., 2013). At 1 day after LPS stimulation, the phagocytic activity of microglia derived from tmTNF^Δ/Δ^ pups was significantly increased compared to microglia derived from tmTNF^wt/wt^ pups, measured as engulfed beads per cell (Figure 7B). Cell area (Figure 7C), perimeter length (Figure 7D), and membrane irregularity (Figure 7E) increased significantly after LPS activation in both tmTNF^wt/wt^ and tmTNF^Δ/Δ^ microglia indicating both tmTNF^wt/wt^ and tmTNF^Δ/Δ^ microglia to take an amoeboid form after LPS activation. Cell morphology was, however, comparable between tmTNF^wt/wt^ and tmTNF^Δ/Δ^ microglia after LPS activation (Figures 7A,C–E).

### Proteomic Characterization of Neurons Derived From tmTNF^Δ/Δ^ and tmTNF^wt/wt^ Mice

In total, 763 proteins with two or more unique peptides were quantified by label free quantification in neurons. Of them, 11 proteins were differentially expressed between tmTNF^Δ/Δ^ and tmTNF^wt/wt^ mice (Supplementary Figure 6). Calcium-activated potassium channel subunit alpha-1 (K_Ca_1.1) was upregulated in tmTNF^Δ/Δ^ mice. HGTD-P that promotes apoptosis in ischemia (Qu et al., 2009) was downregulated in neurons derived from tmTNF^Δ/Δ^ mice suggesting improved neuroprotection.

## Discussion

In the present study, we found that topical administration of the selective solTNF inhibitor XPro1595 decreased infarct volumes 1 and 3 days after pMCAO. Inhibition of TNF by the non-selective solTNF and tmTNF inhibitor etanercept showed no effect. Furthermore, there was no reduction in infarct volume or improvement in functional outcome when TNF inhibitors were delivered by i.c.v. administration, even though the concentrations of the inhibitors in brain tissue were sufficient to neutralize TNF and potentially promote neuroprotection as demonstrated in previous studies using animal models of Alzheimer’s disease (MacPherson et al., 2017).

We have previously shown that topical, but not systemic, administration of XPro1595 decreased lesion volume and improved functional outcome after moderate spinal cord injury (Novrup et al., 2014). Systemic administration of XPro1595 and etanercept had no effect on the infarct volume after pMCAO (Clausen et al., 2014), and systemic etanercept administration had no effect after transient focal cerebral ischemia (Sumbria et al., 2012), indicating that TNF inhibitors have to be administered directly to infarcted brain tissue and not by intracerebroventricular or peripheral routes. The length of treatment may also play a role in suppressing the infarct formation in the acute phase of ischemic stroke. It is known that the infarct volume correlates with functional outcome (Saver et al., 1999; Yoo et al., 2012), and since TNF inhibitors are known to improve functional outcome even without reduction in infarct volume when administered systemically (Clausen et al., 2014), assessment of the functional outcome would have been ideal to evaluate the neuroprotective effects of topical XPro1595 administration. Evaluation of the functional outcome was impossible, however, due to the placement of the mini-osmotic pumps in topically treated animals.

The expression of *Tnf* mRNA in central nervous system has been shown to peak 12 h after pMCAO in mice (Lambertsen et al., 2005, 2009), and TNF protein expression 24 h after pMCAO (Lambertsen et al., 2005, 2009; Clausen et al., 2008). Expression of *Tnf* mRNA in the contralateral hemisphere has been shown to remain at baseline levels after pMCAO (Lambertsen et al., 2009). In permanent focal cerebral ischemia, TNF is believed to exert its neuroprotective effects through TNFR1 (Taoufik et al., 2007; Lambertsen et al., 2009). *Tnfr1* mRNA expression peaks 1–2 days after pMCAO (Lambertsen et al., 2007, 2009), while the protein expression can be detected from 4 to 6 h until 5 days (Botchkina et al., 1997; Lambertsen et al., 2007). The expression of *Tnfr2* increases 1–10 days after pMCAO and reaches its peak 5 days after occlusion (Lambertsen et al., 2007). The protein expression of TNFR2 can be detected 24 h after pMCAO (Botchkina et al., 1997). In our study, topical administration of XPro1595 tended to suppress the expression of TNF protein 1 day after pMCAO, while gene expression was significantly upregulated due to the feedback mechanism by which TNF induces its own gene expression when the amount of available protein is repressed. In addition, inhibition of both solTNF and tmTNF with etanercept downregulated TNFR1 protein levels at day 1. Together with earlier findings that TNFR1 knock-out mice develop larger infarct volumes (Bruce et al., 1996; Gary et al., 1998) and neuroprotection in ischemic stroke is mediated through the TNFR1-NFκB-FLIP(L)-ERK1/2 pathway (Taoufik et al., 2007; Lambertsen et al., 2009; Marques-Fernandez et al., 2013), downregulation of TNFR1 is likely to contribute to the lack of neuroprotection after TNF inhibition with etanercept (Gary et al., 1998; Lambertsen et al., 2009). Given the neuroprotective effect of TNFR2 signaling in our spinal cord injury model (Novrup et al., 2014) and the protective effect of this receptor in chronic, inflammatory conditions such as multiple sclerosis (Brambilla et al., 2011; Gao et al., 2017), we cannot exclude that TNFR2 signaling on microglia may have contributed to the protective effects observed after topical XPro1595 treatment.

TNF is primarily secreted by activated resident microglia and at later time points by infiltrating macrophages after pMCAO (Clausen et al., 2008; Lambertsen et al., 2009). In contrast to earlier studies showing that systemic inhibition of solTNF increased the number of microglia 6 and 24 h after pMCAO (Clausen et al., 2014), flow cytometric analysis of the percentage of microglia and infiltrating macrophages or their CD11b and CD45 MFI values showed no difference between treatment groups. Interestingly, the number of CD45^dim^CD11b^+^ microglia was higher in sham mice treated with either TNF inhibitor when compared to saline-treated mice. In contrast to earlier studies where XPro1595 decreased glial activation in an animal model of Parkinson’s disease (Barnum et al., 2014), it is possible that topical administration of XPro1595 shifted the microglial phenotype without influencing the activation status of the cells. In support of this, *Cx3cr1* mRNA levels were significantly downregulated after 1 and 3 days of topical administration of XPro1595. Also, CX3CR1 deficiency has been shown to be neuroprotective in the early phase after ischemia by promoting microglial polarization to an M2-like phenotype and to decrease microglial activation and neurotoxicity by decreasing the expression of M1-like microglial phenotype that is associated with proinflammatory molecules; all of which lead to smaller infarct volumes (Fumagalli et al., 2013; Tang et al., 2014). XPro1595 also increased the expression of *P2ry12*, a protein related to resting state microglia (Haynes et al., 2006), 1 day after pMCAO when administered topically, suggesting that XPro1595 decreases microglial activation as earlier reported (Barnum et al., 2014; Hsiao et al., 2014; Novrup et al., 2014; MacPherson et al., 2017). Furthermore, the expression of *Trem2* was upregulated 1 and 3 days after pMCAO in mice treated topically with XPro1595. Trem2-knock out mice have altered microglial phagocytosis and larger infarct volumes after cerebral ischemia (Takahashi et al., 2005; Kawabori et al., 2015), supporting earlier studies where XPro1595 was found to promote neuroprotection by improving phagocytosis of dying neurons and cell debris in demyelinated lesions in an animal model of multiple sclerosis (Karamita et al., 2017). Chemokine expression was also altered after treatment with TNF inhibitors.

In the present study, topical administration of XPro1595 increased the relative expression of *Ccl2* mRNA 3 days after pMCAO. CCL2 is necessary for hypoxia-required ischemic tolerance, and upregulation after XPro1595 treatment supports the neuroprotective properties of the selective solTNF inhibitor (Stowe et al., 2012; Wacker et al., 2012). *Cxcl1* mRNA, a proinflammatory granulocyte attracting chemokine expressed by monocytes and granulocytes, was upregulated in etanercept-treated but not XPro1595-treated mice 1 and 3 days after pMCAO. However, there was no difference in the CXCL1 protein levels or in granulocyte infiltration between the treatment groups, contradicting earlier findings where systemic treatment with TNF inhibitors decreased the number of infiltrating granulocytes (Clausen et al., 2014) and TNF deficiency increased the number of infiltrating leukocytes 24 h after pMCAO (Lambertsen et al., 2009).

Intracerebroventricular administration of XPro1595 or etanercept had no effect on the infarct volume nor any beneficial effect on functional outcome despite both drugs being shown to access neural tissue in therapeutically relevant concentrations. In both experimental conditions (i.c.v. versus topical administration), XPro1595 and etanercept were present in more than 1000-fold excess of TNF in the ischemic brain tissue. However, it is possible that despite the penetration of XPro1595 and etanercept, the drugs did not reach their site of action, the penumbra, within the therapeutic window, which in our pMCAO model has been shown to be 6 h (Clausen et al., 2006).

Proteomic characterization of microglia derived from tmTNF^Δ/Δ^ mice revealed that several phagocytosis-related proteins were significantly upregulated compared to proteins from microglia derived from tmTNF^wt/wt^ mice. Microglia derived from tmTNF^Δ/Δ^ mice showed an upregulated expression of 3-MPST, an enzyme protecting cells from oxidative stress by producing redox regulators (Kimura et al., 2017). In addition, 3-MPST regulates hydrogen sulfide production, which has been shown to promote phagocytosis (Dufton et al., 2012), functions as a vasodilator supporting blood flow (Zhao et al., 2001), and promotes angiogenesis (Papapetropoulos et al., 2009). Among the upregulated proteins was HPGDS, which is upregulated in microglia and macrophages after ischemic stroke and is likely to promote microglia/macrophage differentiation to the phagocytic phenotype (Mohri et al., 2003, 2006; Liu et al., 2007). In addition, HPGDS inhibits production of reactive oxygen species and downregulates the expression of the pro-inflammatory cytokine IL-1β through the formation of prostaglandins (Gomez-Abellan et al., 2015). Microglia derived from tmTNF^Δ/Δ^ mice also had an upregulated expression of PYCARD/ASC. PYCARD/ASC deficient mice have impaired lymphocyte migration and are deficient in the number of lymphocytes and dendritic cells in secondary lymphoid organs compromising the normal immune system function (Ippagunta et al., 2011). Upregulation of the microglial PYCARD/ASC could contribute to the maintenance of the innate immune system and the secondary lymphoid organs where tmTNF has shown to be crucial (Ruuls et al., 2001; Alexopoulou et al., 2006). Among the downregulated proteins in tmTNF expressing microglia was MPO, a reactive oxygen species generating enzyme known to be expressed by leukocytes and to increase in cerebral ischemia (Lau and Baldus, 2006; Breckwoldt et al., 2008). Inhibition of MPO increases the proliferation and survival of neurons in animal models of ischemic stroke (Kim et al., 2016). In addition, HGTD-P, that mediates apoptosis in the ischemic brain when activated by hypoxia-inducible factor-1 (Qu et al., 2009), was downregulated in tmTNF expressing microglia.

In order to study whether the up- and downregulation of the phagocytosis-related proteins would affect microglial phagocytosis *in vitro*, we tested phagocytic capacity after 1 day of LPS stimulation. Primary microglia derived from tmTNF^Δ/Δ^ mice showed increased phagocytic activity that most likely can lead to increased clearing of debris thereby promoting neuronal survival in the ischemic area when solTNF is ablated. Earlier studies have demonstrated increased phagocytosis after inhibition of solTNF (Karakantza et al., 2003) and impaired phagocytic ability of TNFR2 deficient microglia (Gao et al., 2017). Since tmTNF preferentially binds to TNFR2, it is likely that phagocytosis is regulated through the tmTNF-TNFR2 pathway. In the present study, we also show that inhibition of solTNF by XPro1595 increased the gene expression of *Trem2*, a receptor that is known to be involved in promoting microglial phagocytosis (Takahashi et al., 2005), an upregulation that was likely to increase microglial phagocytosis in mice treated topically with XPro1595.

This study demonstrates that selective inhibition of solTNF is neuroprotective after pMCAO when administered topically for 1 and 3 consecutive days. Furthermore, topical administration of XPro1595 promotes a phagocytic, microglial phenotype possibly supporting a neurotrophic environment in the ischemic lesion. We suggest that topical administration of XPro1595 has potential to be clinically beneficial in treating patients with ischemic stroke.

## Data Availability

The datasets generated for this study are available on request to the corresponding author.

## Ethics Statement

The Danish Animal Inspectorate under the Ministry of Food and Agriculture (J. No. 2013-15-2934-00924).

## Author Contributions

MY-K, BC, HN, DE, MM, and KL conducted animal surgeries. MD assisted with flow cytometric analyses and helped analyze and interpret data. AS and PT-J performed proteomics. RB performed experiments and helped draft the manuscript. DS provided the XPro1595 and gave useful input to the drafting of the manuscript. MY-K, BC, and KL performed experiments, interpreted results, performed statistical analyses, and wrote the manuscript. KL conceived the study. All authors read and approved the final manuscript.

## Conflict of Interest Statement

DS was an employee of Xencor and holds stock and stock options in the company. The remaining authors declare that the research was conducted in the absence of any commercial or financial relationships that could be construed as a potential conflict of interest.

## Footnotes

1 http://proteomecentral.proteomexchange.org

2 http://string-db.org

3 https://github.com/HA-Lab/PuntoMorph

## Abbreviations

3-MPST: 3-mercaptopyruvate sulfurtransferase
Arg1: arginase 1
CCL2: C-C motif chemokine ligand 2
CV: coefficient of variation
CXCL1: C-X -C motif chemokine ligand 1
CXCL10: C-X -C motif chemokine ligand 10
Cx3cr1: C-X 3-C motif chemokine receptor 1
FBS: fetal bovine serum
FDR: false discovery rate
FMO: fluorescence minus one
HBSS: Hanks’ Balanced Salt Solution
HGTD-P: human growth and transformation-dependent protein
HPGDS: hematopoetic prostaglandin D synthase
HPRT1: hypoxanthine phosphoribosyltransferase 1
i.c.v.: intracerebroventricular
IL-1 β: interleukin-1 β
INOS: inducible nitric oxide synthase
LPS: lipopolysaccharide
MCA: middle cerebral artery
MECP: methyl-CgP-binding protein 2
MFI: mean fluorescent intensity
MPO: myeloperoxidase
PBS: phosphate buffered saline
pMCAO: permanent middle cerebral artery occlusion
P2ry12: P2Y purinoceptor 12
PYCARD: apoptosis-associated speck-like protein containing CARD
RIN: RNA integrity factor
RT-PCR: reverse transcriptase-polymerase chain reaction
s.c.: subcutaneously
solTNF: soluble tumor necrosis factor
SSC: side scatter
TACE: tumor necrosis factor alpha converting enzyme
tmTNF: transmembrane tumor necrosis factor
TNF: tumor necrosis factor
TNFR1: tumor necrosis factor 1
TNFR2: tumor necrosis factor 2
Trem2: triggering receptor expressed on myeloid cells-2
UPLC-MS/MS: ultra-performance liquid chromatography-tandem mass spectrometry.
