# Supplementary material for: Topical Administration of a Soluble TNF Inhibitor Reduces Infarct Volume After Focal Cerebral Ischemia in Mice
Source: Front Neurosci. 2019 Aug 7;13:781. doi: 10.3389/fnins.2019.00781 (PMC6692878; doi:10.3389/fnins.2019.00781)
Supplement: Supplementary file 1 [file Data_Sheet_1.docx]

**Topical administration of a soluble TNF inhibitor reduces infarct volume after focal cerebral ischemia in mice**

Minna Yli-Karjanmaa^†1^, Bettina Hjelm Clausen^†1,2^, Matilda Degn^3^, Hans Gram Novrup^1^, Ditte Gry Ellman^1^, Peter Toft-Jensen^1^, David E. Szymkowski^4^, Allan Stensballe^5^, Morten Meyer^1,2^, Roberta Brambilla^1,2,6^, Kate Lykke Lambertsen*^1,2,7^

^1^Department of Neurobiology Research, Institute of Molecular Medicine, University of Southern Denmark, Odense, Denmark

^2^BRIDGE – Brain Research Inter-Disciplinary Guided Excellence, Department of Clinical Research, University of Southern Denmark, Odense, Denmark

^3^Pediatric Oncology Laboratory, Department of Pediatrics and Adolescent Medicine, University Hospital Rigshospitalet, Copenhagen, Denmark

^4^Xencor Inc, Monrovia, CA, USA

^5^Department of Health Science and Technology, University of Aalborg, Aalborg, Denmark

^6^The Miami Project to Cure Paralysis, University of Miami Miller School of Medicine, Miami, FL, USA

^7^Department of Neurology, Odense University Hospital, Odense, Denmark


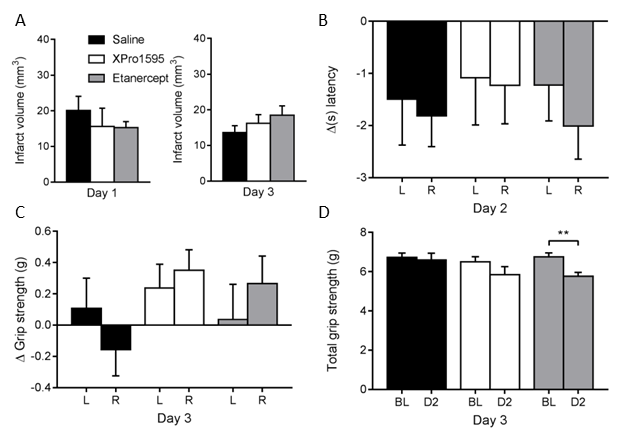


**Supplementary Figure 1. Infarct volume and functional outcome after i.c.v. administration**. **(A)** Infarct volume 1 and 3 days after i.c.v administration of TNF inhibitors n(day 1)= 3-5/group, n(day 3)=15-17/group. **(B)** Withdrawal latency after nociceptive stimuli measured by Hargreaves test two days after pMCAO; n=13-17/group. **(C)** Grip strength test of neuromuscular function measured as asymmetry between the right and left side three days after pMCAO; n=16-18/group. **(D)** Grip strength test of neuromuscular function measured as total grip strength of both paws three days after pMCAO; n=16-17/group. **p≤0.01, Student’s paired t-test.


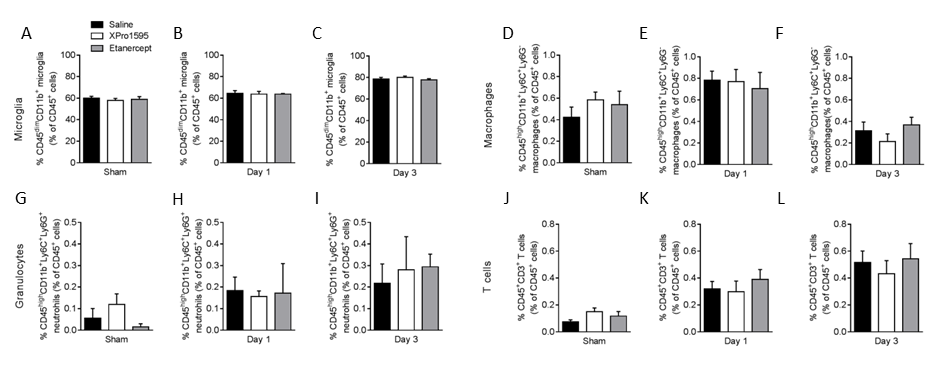


**Supplementary Figure 2. Flow cytometric analysis of contralateral hemispheres in sham and pMCAO mice after topical saline, XPro1595, or etanercept treatment. (A-C)** Changes in CD45^dim^CD11b^+^ microglia presented as % of CD45^+^ cells after sham surgery or 1 or 3 days after pMCAO. **(D-F)** Changes in CD45^high^CD11b^+^Ly6C^+^Ly6G^-^ macrophages presented as % of CD45^+^ cells after sham surgery or 1 or 3 days after pMCAO. **(G-I)** Changes in CD45^high^CD11b^+^Ly6C^+^Ly6G^+^ granulocytes presented as % of CD45^+^ cells after sham surgery or 1 or 3 days after pMCAO. **(J-L)** Changes in CD3^+^ T cells presented as % of CD45^+^ cells after sham surgery or 1 or 3 days after pMCAO. n(sham)=4-5/group; n(day 1)=4-5/group; n(day 3)=5/group.


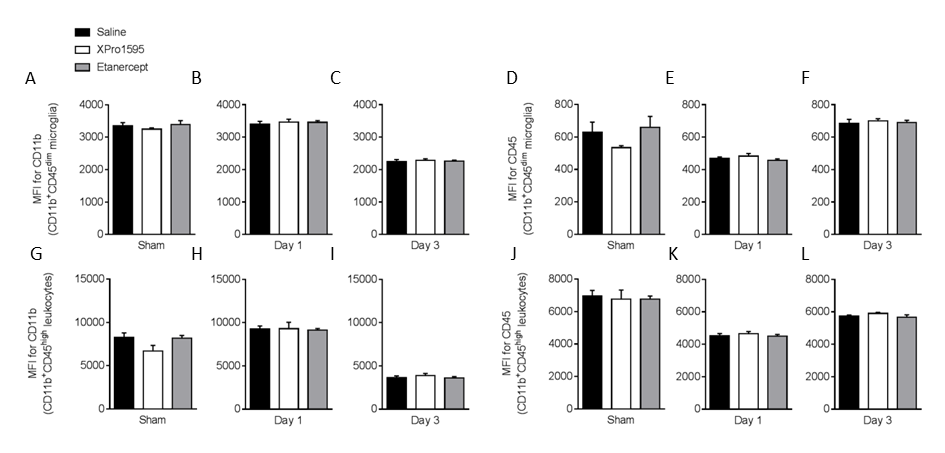


**Supplementary Figure 3. Mean fluorescence intensity (MFI) of CD11b and CD45 in the ipsilateral cortex of mice treated topically with saline, XPro1595, or etanercept. (A-C)** MFI for CD11b of CD11b^+^CD45^dim^ microglia after sham surgery or 1 or 3 days after pMCAO. **(D-F)** MFI for CD45 of CD11b^+^CD45^dim^ microglia after sham surgery or 1 or 3 days after pMCAO. **(G-I)** MFI for CD11b of CD11b^+^CD45^high^ leukocytes after sham surgery or 1 or 3 days after pMCAO. **(J-L)** MFI for CD45 of CD11b^+^CD45^high^ leukocytes after sham surgery or 1 or 3 days after pMCAO. N(sham)=4-5/group; n(day 1)=4-5/group; n(day 3)=5/group.


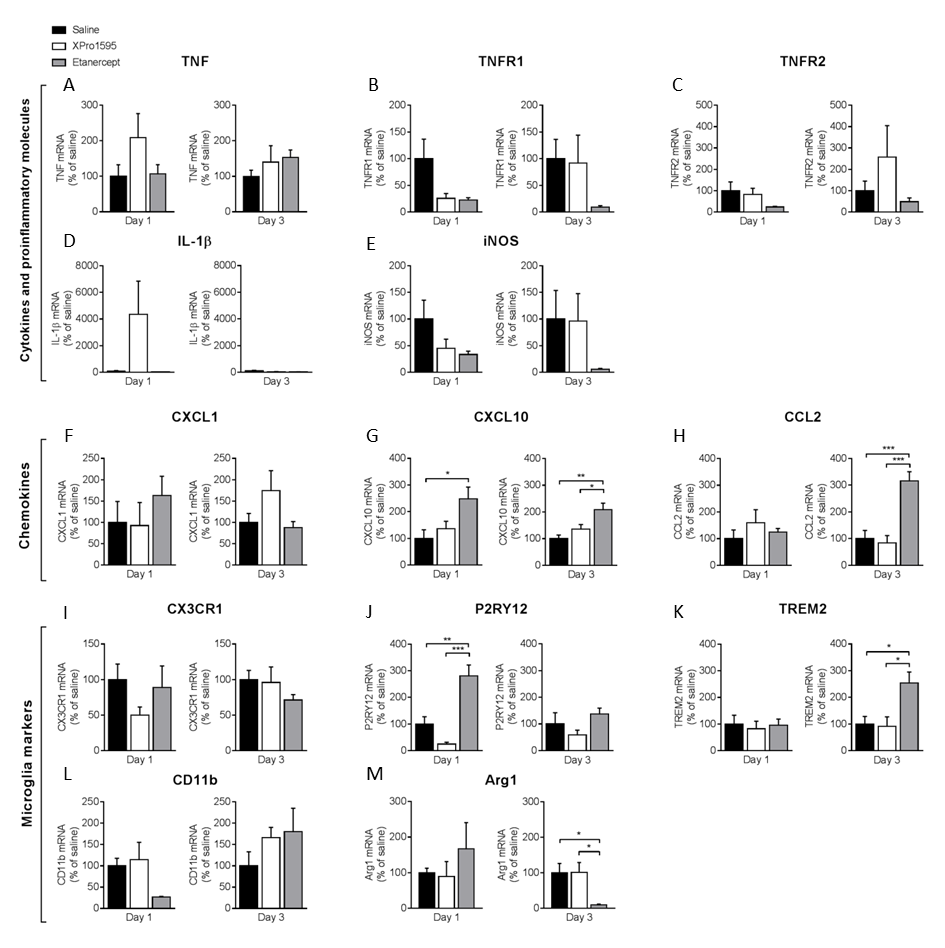


**Supplementary Figure 4. Inflammatory gene profile 1 and 3 days after pMCAO in mice treated i.c.v. with saline, XPro1595, or saline.** mRNA expression of cytokines, pro-inflammatory molecules, chemokines, and microglial markers presented as % of gene expression in saline-treated animals **(A-M). (A)** Expression of *Tnf* mRNA. **(B)** Expression of *Tnfrsf1a* (TNFR1) mRNA. **(C)** Expression of *Tnfrsf1b* (TNFR2) mRNA. **(D)** Expression of *Il1β* mRNA. **(E)** Expression of *iNOS* mRNA. **(F)** Expression of *Cxcl1* mRNA. **(G)** Expression of *Cxcl10* mRNA. **(H)** Expression of *Ccl2* mRNA. **(I)** Expression of *Cx3cr1* mRNA. **(J)** Expression of *P2ry12* mRNA. **(K)** Expression of *Trem2* mRNA. **(L)** Expression of *Cd11b* mRNA. **(M)** Expression of *Arg1* mRNA. N(day 1)=3-5/group, n(day 3)=4-6/group;*p≤0.05, **p≤0.01, ***p≤0.001; one-way ANOVA followed by Tukey’s *post hoc* test.


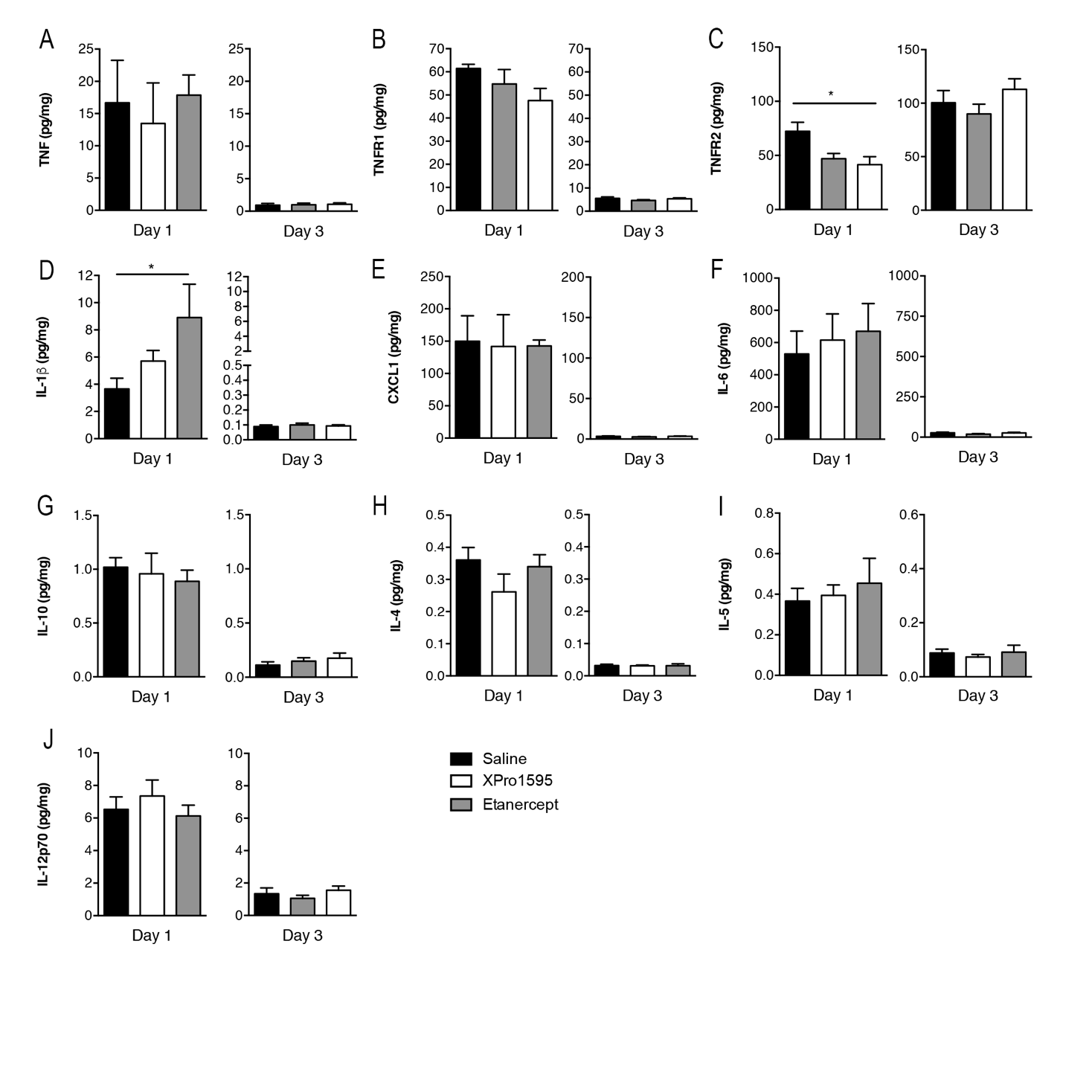


**Supplementary Figure 5. Cytokine and receptor protein expression in brain tissue 1 and 3 days after pMCAO in mice treated i.c.v. with saline, XPro1595, or etanercept. (A)** Expression of TNF. **(B)** Expression of TNFR1. **(C)** Expression of TNFR2. **(D)** Expression of IL-1β. **(E)** Expression of Cxcl1. **(F)** Expression of IL-6. **(G)** Expression of IL-10. **(H)** Expression of IL-4. **(I)** Expression of IL-5. **(J)** Expression of IL-12p70. N=3-6/group, **p≤0.01; one-way ANOVA with Tukey’s *post hoc* test.


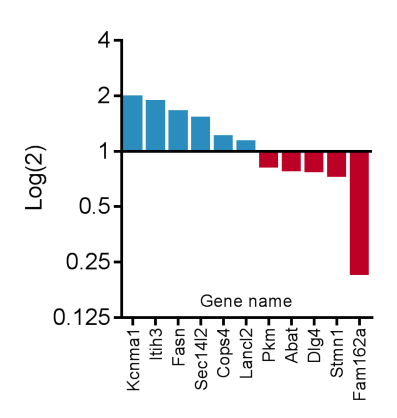


**Supplementary Figure 6. Proteomics of neurons from tmTNF^Δ/Δ^ and tmTNF^wt/wt^ mice.** Up- and downregulated proteins in neurons from tmTNF^Δ/Δ^ and tmTNF^wt/wt^ mice. N(tmTNF^wt/wt^)= 4, n(tmTNF^Δ/Δ^)= 6.
